# Blind haste: As light decreases, speeding increases

**DOI:** 10.1371/journal.pone.0188951

**Published:** 2018-01-03

**Authors:** Emanuel de Bellis, Michael Schulte-Mecklenbeck, Wernher Brucks, Andreas Herrmann, Ralph Hertwig

**Affiliations:** 1 Institute for Customer Insight, University of St. Gallen, St. Gallen, Switzerland; 2 Department of Business Administration, University of Bern, Bern, Switzerland; 3 Center for Adaptive Rationality, Max Planck Institute for Human Development, Berlin, Germany; 4 Traffic Division of the City of Zurich, Zurich, Switzerland; Politecnico di Torino, ITALY

## Abstract

Worldwide, more than one million people die on the roads each year. A third of these fatal accidents are attributed to speeding, with properties of the individual driver and the environment regarded as key contributing factors. We examine real-world speeding behavior and its interaction with illuminance, an environmental property defined as the luminous flux incident on a surface. Drawing on an analysis of 1.2 million vehicle movements, we show that reduced illuminance levels are associated with increased speeding. This relationship persists when we control for factors known to influence speeding (e.g., fluctuations in traffic volume) and consider proxies of illuminance (e.g., sight distance). Our findings add to a long-standing debate about how the quality of visual conditions affects drivers’ speed perception and driving speed. Policy makers can intervene by educating drivers about the inverse illuminance‒speeding relationship and by testing how improved vehicle headlights and smart road lighting can attenuate speeding.

## Introduction

In 2015, as many as 9,557 lives were lost in speeding-related accidents in the US alone, with an estimated annual economic cost to society amounting to USD 52 billion [1]. Extensive research has investigated the causes of and factors contributing to speeding, including factors beyond reach of the driver, such as social norms or inclement weather [2,3]. Nevertheless, it is unclear how the quality of visual conditions, a ubiquitous environmental factor, affects drivers’ speeding behavior. In theory, every driver knows one of the key principles of road safety: If visual conditions are poor, reduce your speed. In practice, however, drivers appear not to slow down enough to counteract the higher risks associated with adverse visual conditions [2,4].

Perception research has uncovered a range of biases that influence humans’ motion perception [5–7], many of which depend on the prevailing visual conditions. For instance, drivers’ ability to recognize non-illuminated objects that necessitate a reduction in speed (e.g., crossroads or pedestrians) is substantially impaired under low contrast, when the difference in brightness between an object and its background is reduced [8]. Although ambient vision (which is of primary importance in guiding locomotion) remains effective under low contrast, focal vision (responsible for visual recognition) is severely impaired, resulting in overconfidence of drivers in the dark [8,9]. This perceptual account is complemented by the inferential nature of human perception. The perceived distance of an object is inferred from, among other cues, its contrast: the higher the contrast of an object, the closer it appears to be [10]. Applying these findings to traffic behavior, we can conclude that drivers operating under adverse visual conditions may not only take longer to recognize objects but also overestimate their distance [11–14], as a result of which deceleration is delayed.

Other lines of research suggest that drivers’ perplexing response to impaired visual conditions is not delayed deceleration, but acceleration. According to the Thompson effect, a classic empirical regularity in vision research, the perceived speed of moving objects is underestimated when contrast is reduced [15–17]. Similar effects have been observed in functional imaging studies at lower levels of contrast, with results showing that these estimation biases arise in the earliest visual cortical regions [18]. Do these experimental findings generalize to actual driving? Driving simulation studies showed that participants in computer-generated driving simulations not only perceived foggy (vs. clear) scenes to move more slowly [19] but also “drove” faster than a given target speed in simulated fog [20]. This finding was replicated using filmed footage of actual traffic situations [21,22]. However, these studies have been criticized for representing a poor model of motion perception in three-dimensional environments, because contrast was reduced uniformly and independently of distance. Research employing more ecologically valid fog simulations [23,24] or examining reduced levels of luminance (i.e., the amount of light emitted or reflected from a particular surface) [25] has found that drivers overestimate their speed—an estimation bias that prompts them to decelerate.

One way to shed light on these contradictory findings is to step outside the laboratory and investigate the effects of visual conditions in real traffic [26]. A study that investigated actual driving behavior on a closed road course (with uniformly reduced contrast and obstructed view of the speedometer) found a reduction in speed when contrast was reduced [27]. Two field studies by Bassani and colleagues with a total of 17,444 observations provide more detailed insights. One study found that during daytime operating speeds increased as illuminance increased, whereas “speeds are higher at nighttime even though in darkness the illuminance values are lower than daytime” [28]. A follow-up study specified that as illuminance increased both average speeds and deviations from the mean increased [29]. However, models that only contained sunny or cloudy conditions showed a negative relationship between illuminance and average speeds. Taken together, these findings do not offer an unequivocal picture with regard to how light and driving speed interact. In our study, we attempted to determine which of the above contradictory findings could be observed in real traffic with all its distinctive environmental and driver-related factors, including other road users. To this end, we used a vastly larger sample (over 1.2 million observations) than previous studies have used. Second, rather than investigating deviations from various target speeds, the standard measure in many experimental studies, we focused on the prevalence of speeding (i.e., exceeding the legal speed limit) as a function of the environment. Third, we extended research on contrast and luminance by investigating the effects of illuminance—a ubiquitous variable that policy makers are able to influence by environmental design (e.g., road lighting).

## Method

Our analyses are based on a large dataset of hidden speed measurements, which were used to create a speeding index. To examine the illuminance‒speeding relationship, we regressed the speeding index on hourly matched illuminance data. In addition, we controlled for factors that are known to influence speeding (e.g., fluctuations in traffic volume), examined proxies of illuminance (e.g., sight distance), and performed a range of robustness checks.

### Speeding dataset

For city planning purposes, the Traffic Division of the City of Zurich regularly performs speed measurements throughout Zurich. With a population of 400,000, Zurich is Switzerland’s largest city, recording approximately 600,000 daily vehicle movements across the city borders [30]. The use of hidden radar systems means that drivers are unaware of the measurements, and they are not prosecuted for violating the speed limit. Over the study period, the radar systems were installed for on average eight consecutive days (*M* = 7.7, *SD* = 4.2) per location and measured each passing vehicle’s driving speed round the clock. Measurements took place on straight road parts free of potential interference (i.e., as far away as possible from crossroads, priority rules, traffic lights, pedestrian crossings, and private exits; see S1 Table for a detailed description of each measurement point). The dataset comprised 1,220,359 vehicle movements, collected in 71 urban Zurich roads in both 30 km/h and 50 km/h zones between May 31, 2007, and August 24, 2009. The measurements were allotted to 5 km/h speed brackets for each hour. To analyze the illuminance‒speeding relationship, we calculated a speeding index by dividing the number of vehicles exceeding the speed limit by the total number of vehicles (per road and hour), thus arriving at an index ranging from 0 (no speeding) to 100 (all vehicles exceeded the speed limit):

$$Speeding\;Index=\frac{N_{speeding}}{N_{total}}\times 100$$

In line with local police regulations, we defined speeding as exceeding the speed limit by more than 5 km/h [31]. For instance, drivers in a 50 km/h zone were considered to speed if their driving speed was 56 km/h or higher. Three portable traffic monitors were used to measure vehicle speed (manufacturer and share of reported measurements are reported in parentheses): (1) the KV Laser (Sodi Scientifica SpA; 25%), which is based on laser technology without external sensors; (2) the LOTOS system (CRVM; 33%), also based on laser technology; and (3) the Radar Traffic Recorder (RTR; Multanova AG; 42%), based on radar technology. S1 and S2 Figs show the location of the monitored roads across Zurich and daily fluctuations in speeding, respectively.

### Meteorological variables

Zurich covers an area of 91.9 km^2^, has a maximum north‒south extension of 12.7 km, and a maximum east‒west extension of 13.4 km [32]. Meteorological variables were measured either at Zurich downtown or at Zurich airport, approximately 8 km north of that. The data were obtained from the Swiss Federal Office of Meteorology and Climatology (https://gate.meteoswiss.ch/idaweb) and the Swiss Federal Office for the Environment (www.bafu.admin.ch). Local sunrise and sunset times were consulted to examine day/night differences. Table 1 provides a detailed overview of the variables we analyzed; S3 Fig shows daily fluctuations in illuminance, our key independent variable.

**Table 1:** Overview of variables.

Variable	Description	Aggregation/ Transformation	Unit	Location	Descriptive Statistics^d^			
					*M*	*SD*	*Min*	*Max*
Traffic volume^a^	Total number of passing vehicles per road and hour	Summed per hour and logarithmized	# of vehicles	71 roads in Zurich	44.26	61.34	1.00	640.00
Speeding index^a^	Proportion of vehicles exceeding the speed limit per road and hour (based on speed variable)	N(speeding) / N(total) × 100	% of vehicles	71 roads in Zurich	15.62	22.68	0.00	100.00
Illuminance^b^	Luminous flux incident on a surface per unit area	Averaged per hour and logarithmized	lx	Zurich airport / Zurich east	22930	35909	0	218800
Global irradiance^b^	Electromagnetic radiation	Averaged per hour and logarithmized	W/m^2^	Zurich airport	177.50	244.24	1.00	1008.00
Sunshine duration^b^	Cumulative time of direct irradiance from the sun (> 120 W/m^2^)	Summed per hour	min	Zurich airport	14.09	22.90	0.00	60.00
Sight distance^b^	Maximal horizontal distance at which an object or light source can be clearly discerned	None (measured at 3-hour intervals)	km	Zurich airport	66.60	15.71	0.00	89.00
Particulate air pollution^c^	Microscopic solid or liquid matter	Averaged per hour and logarithmized	μg/m^3^	Zurich down-town	17.67	10.84	0.00	186.20
Precipita-tion^b^	Condensation of atmospheric water vapor	Summed per hour and logarithmized	mm	Zurich airport	0.12	0.69	0.00	18.70
Fog^b^	Low-lying clouds	None (measured at 3-hour intervals)	Binary (present/absent)	Zurich airport	Fog present: 1.24% Fog absent: 98.76%			

Notes: Sources for the reported data are

(a) Traffic Division of the City of Zurich, Switzerland.

(b) Swiss Federal Office of Meteorology and Climatology, and

(c) Swiss Federal Office for the Environment.

(d) To facilitate interpretation, we show non-transformed values; *M* indicates the mean, *SD* the standard deviation, *Min* the minimum, and *Max* the maximum.

## Results

### The illuminance–speeding relationship

**Fig 1:**
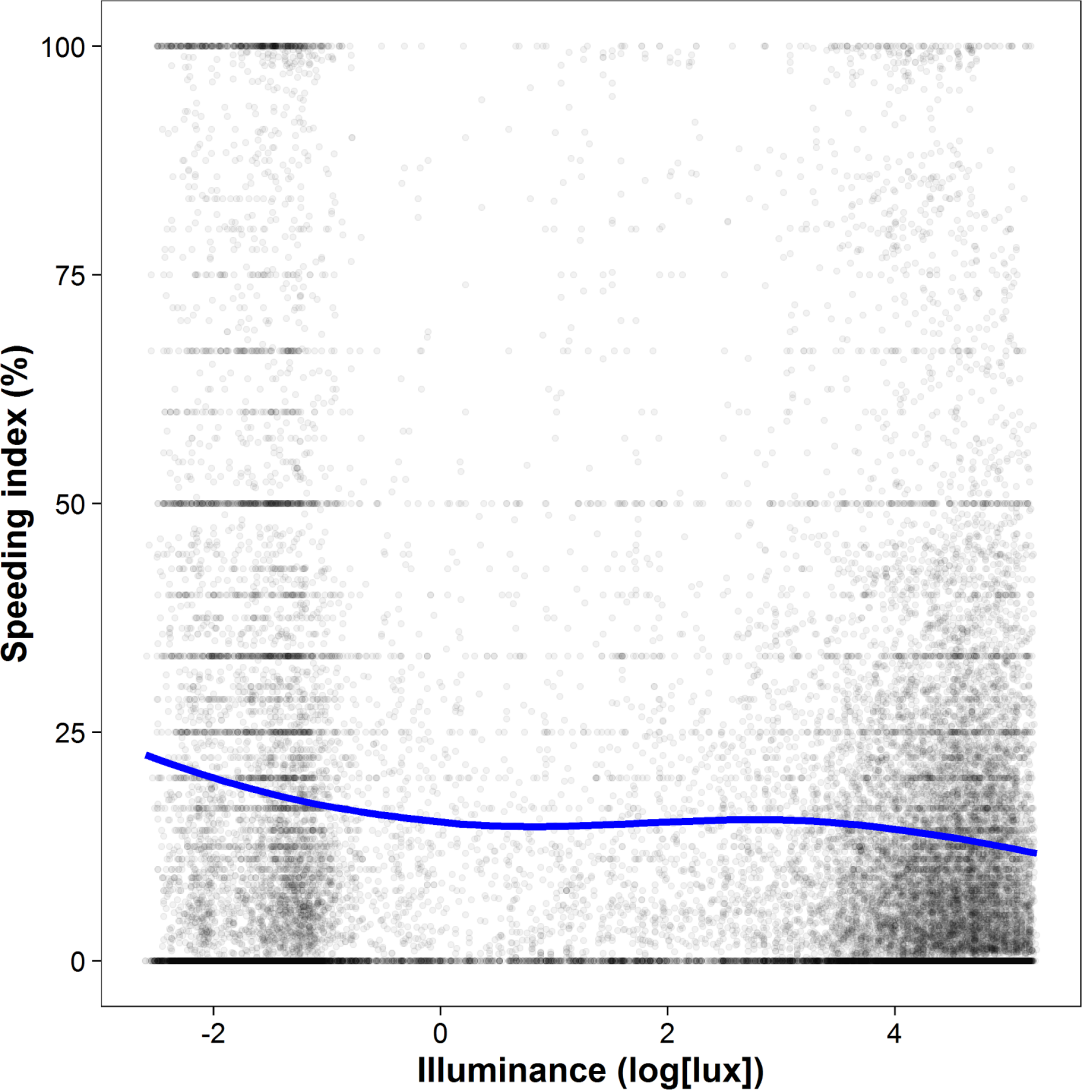
The inverse illuminance‒speeding relationship. Each data point depicts a road at a given hour and represents 1 to 640 vehicles depending on traffic volume. The blue line represents a loess curve (i.e., local polynomial regression fitting). Results show a negative relation between illuminance (log[lux]) and speeding at low and high illuminance levels, but not at intermediate illuminance levels. Note that removing outliers (e.g., data points with a speeding index of 0 and 100) or less frequented roads (e.g., data points with a traffic volume of 5 and below) did not change the overall pattern of results.

Unless otherwise specified, we employed linear OLS regression analyses to examine focal effects, with illuminance as the independent variable, the speeding index as the dependent variable, and traffic volume as a covariate. This basic model yielded an inverse relationship between illuminance and the speeding index (*b* = −1.24, *t*(27465) = −23.88, *p* < .001, partial *η*^2^ = .02), indicating that a reduction in illuminance was associated with an increase in speeding behavior. A reduction in illuminance of 100 lux was thus associated with an increase in speeding of 0.59 percentage points. In other words, the likelihood of speeding (i.e., observations above the median of the speeding index) increased significantly when illuminance was low (i.e., observations below the median of illuminance; odds ratio = 1.08; 95% confidence interval = 1.03 to 1.13). A cubic polynomial regression fitted the pattern obtained in Fig 1 best, with a significant linear (*b* = −586.41, *t*(27463) = −24.06, *p* < .001), quadratic (*b* = 74.32, *t*(27463) = 3.32, *p* < .001), and cubic term (*b* = −169.74, *t*(27463) = −7.60, *p* < .001). The cubic function explained significantly more variance than a quadratic function (*F* = 57.73, *p* < .001) and was not outperformed by a quartic function (*F* = 0.60, *p* > .25).

**Fig 2:**
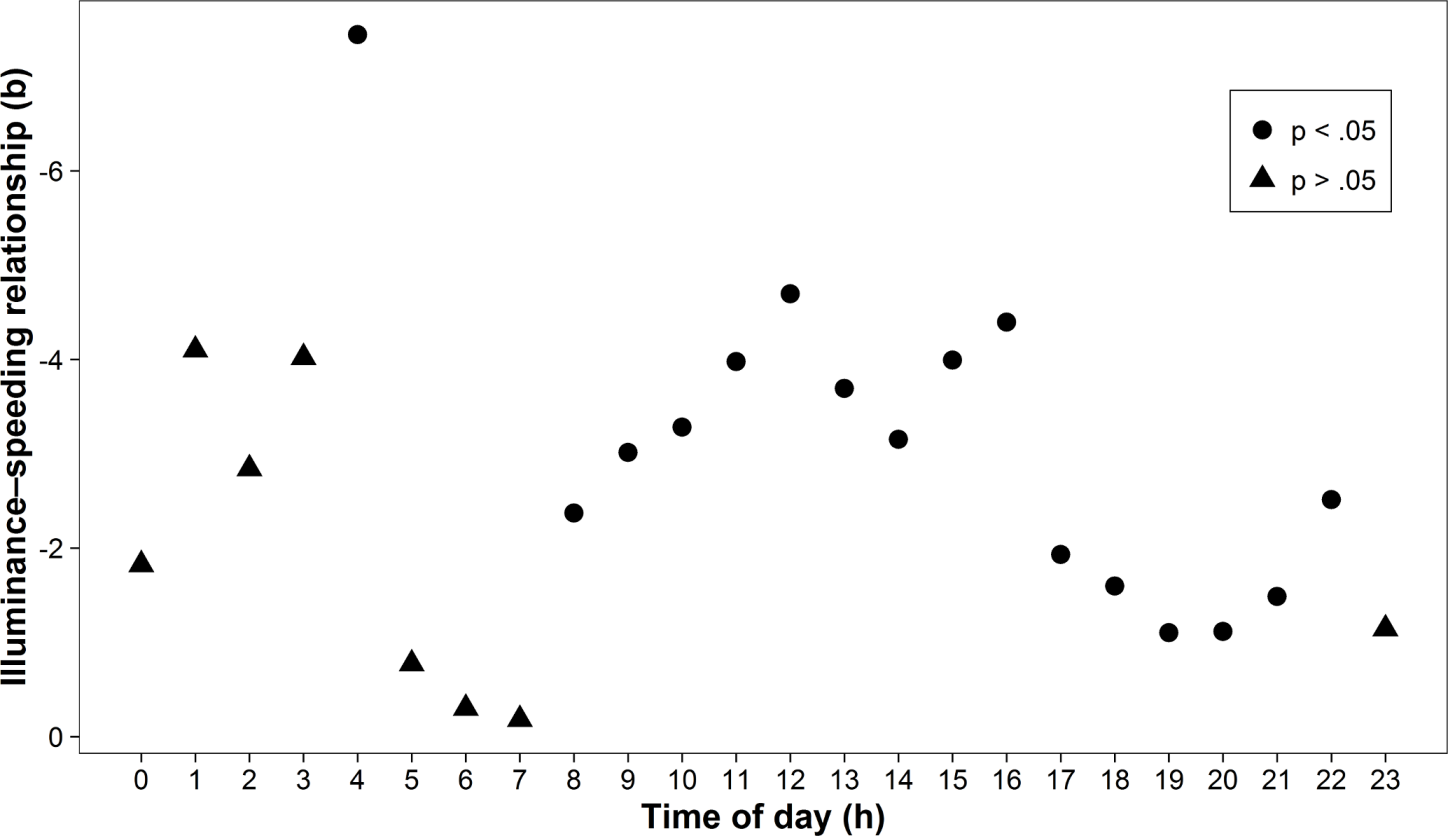
The illuminance‒speeding relationship by time of day. We calculated separate regression analyses for each hour of the day. The y-axis indicates non-standardized regression coefficients and is reversed for ease of interpretation (i.e., higher numbers indicate a stronger negative relationship). Time of day corresponds to the local time (UTC+1 adjusted for daylight saving time). The illuminance‒speeding relationship was particularly strong after noon and was (with one exception) non-significant at night, when traffic volume reaches its daily minimum.

**Fig 3:**
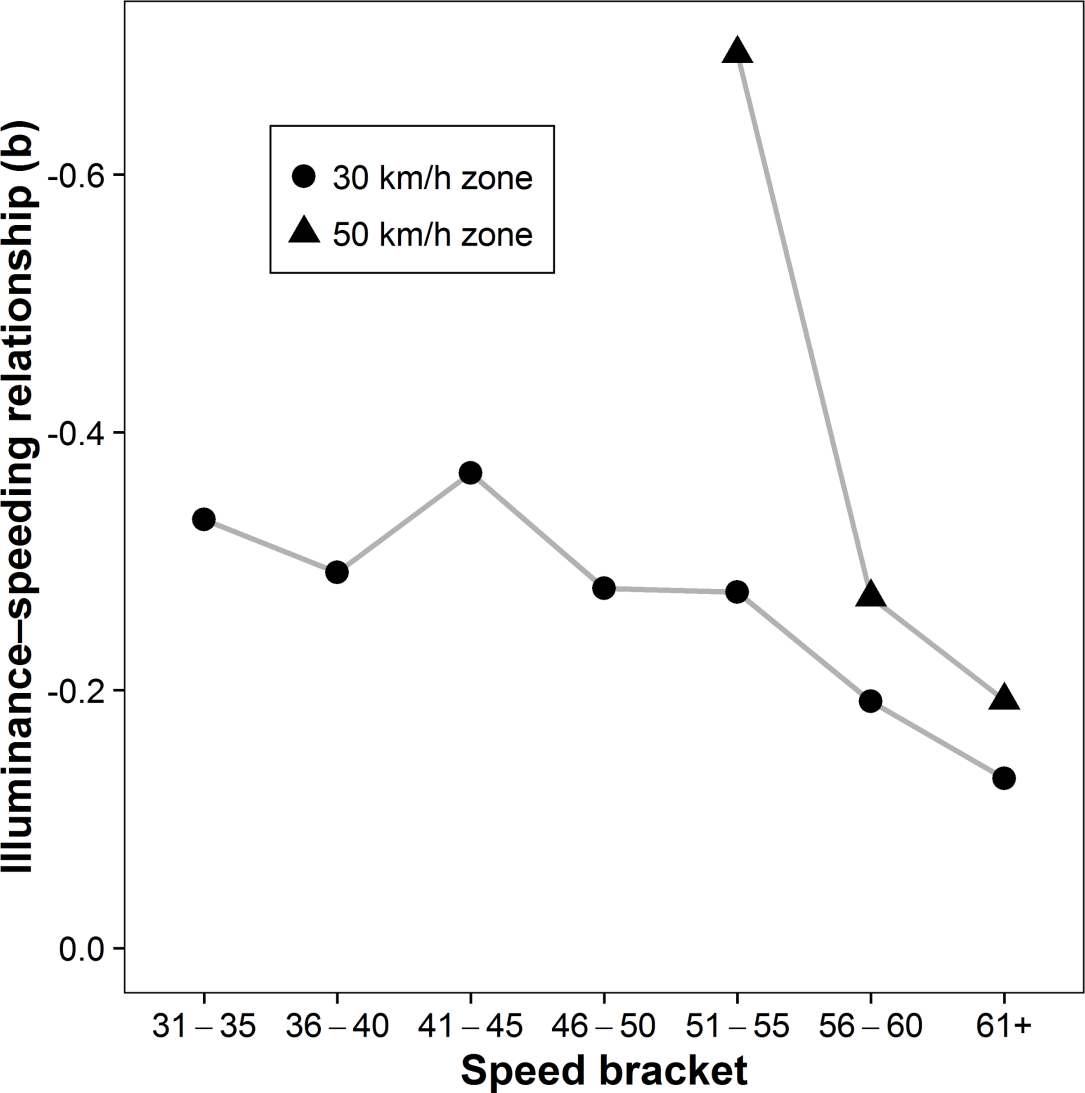
The illuminance‒speeding relationship across speed brackets. For both 30 km/h and 50 km/h zones, we calculated separate regression analyses for each speed bracket. The y-axis indicates non-standardized regression coefficients and is reversed for ease of interpretation (i.e., higher numbers indicate a stronger negative relationship). Across speed brackets, we found support for the inverse illuminance‒speeding relationship. While the strength of the relationship declined with increasing speeding rates, all regression coefficients were significant at *p* < .001.

The pattern in Fig 1 also indicates that the association between illuminance and speeding may not be constant across the 24-hour cycle. In order to analyze this variability further, we calculated separate regression analyses for each hour of the day. The relationship proved to be constantly negative and significant for every hour between 8 am and 10 pm (all *b*s < −1.10, all *p*s < .05), a period accounting for 84.4% of daily traffic volume, and negative but mostly non-significant between 11 pm and 7 am (Fig 2). In addition, we analyzed the *degree* of speeding instead of using a dichotomous measure (below or above the speeding threshold). Separate regression analyses performed for each speed bracket (e.g., driving 31−35 km/h in a 30 km/h zone) showed that the inverse relationship between illuminance and speeding was significant across speed brackets, but that its strength declined at higher levels of speeding (Fig 3).

### Robustness checks

**Fig 4:**
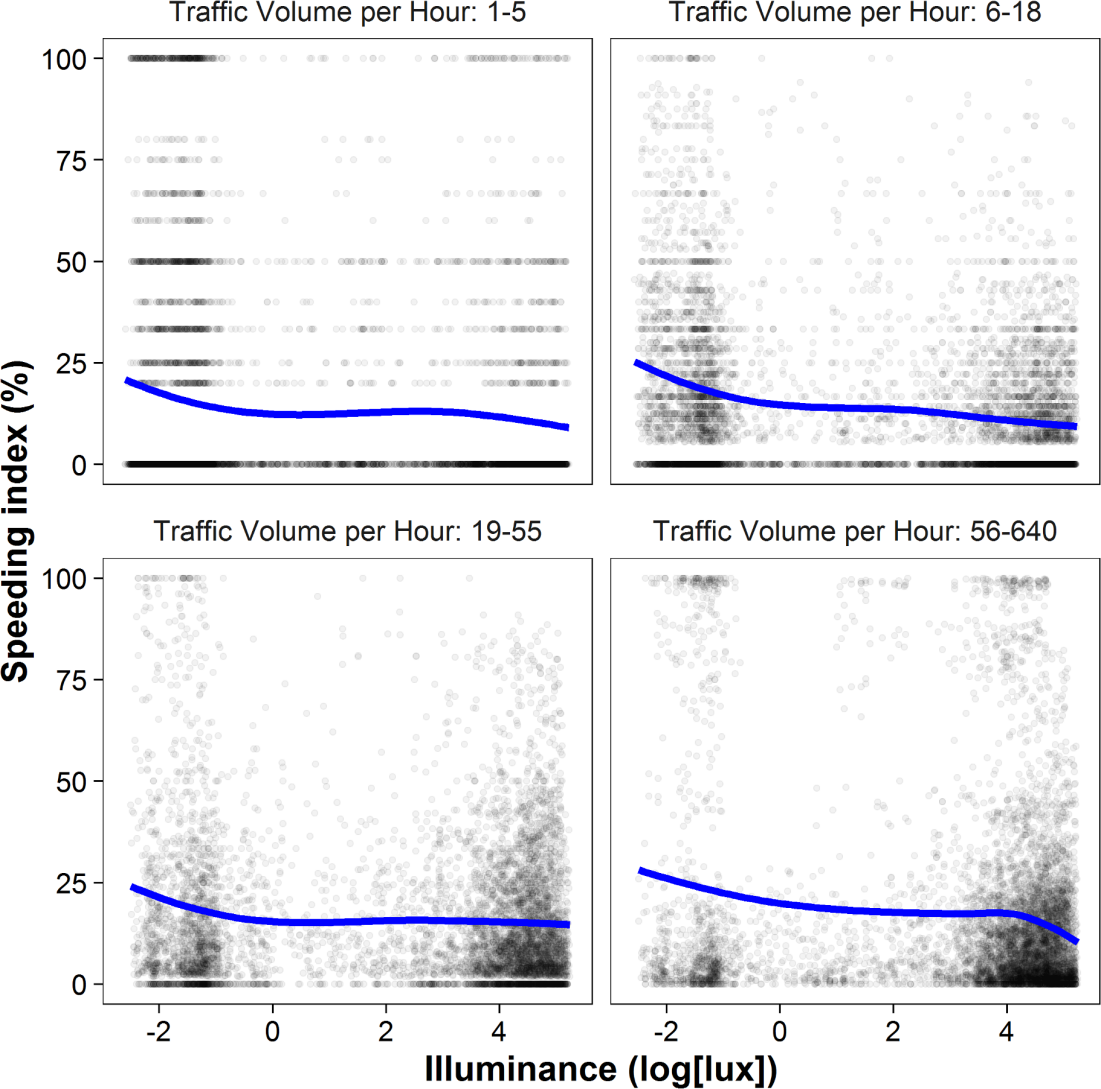
The illuminance‒speeding relationship by traffic volume. This figure depicts the relationship between illuminance and speeding as in Fig 1, but the data are split by the traffic volume of the respective road per hour. We chose four brackets, each containing a similar number of measurements: 1‒5 vehicles per hour (6497 measurements), 6‒18 (6917), 19‒55 (7012), and 56‒640 (7147). The fitted line shows some variation across the four panels, but they all point to an inverse illuminance‒speeding relationship. Regression analyses for each bracket proved significant: 1‒5 vehicles per hour (*b* = −0.94, *t*(6469) = − 7.39, *p* < .001, partial *η*^2^ = .01), 6‒18 (*b* = −1.56, *t*(6890) = −19.88, *p* < .001, partial *η*^2^ = .05), 19‒55 (*b* = −0.65, *t*(6985) = −7.65, *p* < .001, partial *η*^2^ = .01), and 56‒640 (*b* = −1.52, *t*(7116) = −11.85, *p* < .001, partial *η*^2^ = .02).

The inverse illuminance‒speeding relationship proved robust across less frequented and highly frequented roads (Fig 4), in 30 km/h and 50 km/h zones, across urban and rural areas, during the day and at night, on weekdays and at weekends, across seasons (Table 2), and when controlling for the distance to the closest crossroad (*b* = −1.38, *p* < .001, *t*(26412) = −25.99, partial *η*^2^ = .02; S1 Table). The relationship persisted when we used a hard speeding cut-off, considering speeds of 31 (51) km/h or faster in a 30 (50) km/h zone as speeding (*b* = −1.87, *p* < .001, *t*(24009) = −26.19, partial *η*^2^ = .03 for 30 km/h zones and *b* = −1.35, *t*(3453) = −14.03, *p* < .001, partial *η*^2^ = .05 for 50 km/h zones). Analysis of the illuminance‒speeding relationship for each type of traffic monitor revealed slight variations in results, but the overall picture was consistent: Illuminance was negatively related to speeding as measured by the KV Laser (*b* = −1.59, *t*(3532) = −12.80, *p* < .001, partial *η*^2^ = .04), the LOTOS system (*b* = −0.43, *t*(5389) = −4.37, *p* < .001, partial *η*^2^ = .004), and the RTR (*b* = −1.12, *t*(8400) = −10.81, *p* < .001, partial *η*^2^ = .01). The use of different measurement systems reduces the probability of systematic measuring errors and therefore speaks for the robustness of the results obtained.

**Table 2:** Robustness checks for the illuminance‒speeding relationship.

	(a)	(b)
Overall	−1.241** (0.052)	−0.854** (0.048)
Less frequented roads (below median)	−1.293** (0.078)	−1.191** (0.076)
Highly frequented roads (above median)	−1.172** (0.070)	−0.979** (0.069)
30 km/h zone	−1.538** (0.057)	−0.911** (0.053)
50 km/h zone	−0.689** (0.073)	−0.443** (0.065)
Urban areas	−1.170** (0.056)	−0.722** (0.052)
Transition urban/rural areas	−0.956** (0.111)	−0.968** (0.102)
Rural areas	−3.229** (0.284)	−1.472** (0.286)
Day (sunrise to sunset)	−2.146** (0.292)	−1.991** (0.294)
Night (sunset to sunrise)	−1.639** (0.157)	−1.124** (0.152)
Noon (12.00 to 12.59 pm)	−4.697* (1.727)	−5.211* (1.727)
Weekdays (Monday to Friday)	−1.382** (0.064)	−0.947** (0.058)
Weekends and statutory holidays	−0.977** (0.088)	−0.654** (0.084)
Spring (March to May)	−1.249** (0.097)	−0.939** (0.091)
Summer (June to August)	−0.698** (0.079)	−0.639** (0.066)
Autumn (September to November)	−1.143** (0.100)	−0.555** (0.095)
Winter (December to February)	−0.923** (0.234)	−0.792** (0.212)

Reported results are non-standardized coefficients of the illuminance‒speeding relationship (linear regression models) with standard errors in parentheses.

(a) The speeding index is regressed on illuminance and traffic volume (both logarithmized).

(b) The second model does not control for traffic volume.

Significance levels

*p < .01.

**p < .001.

In addition to the reported linear and polynomial regressions, we performed a linear mixed-effects analysis using the lme4 R package [33] to account for the random effects structure of the dataset. As fixed effects, we entered illuminance and traffic volume (both logarithmized and without interaction term) into the model. As random effects, we entered an intercept for road, as well as a by-road random slope for the effect of illuminance. As in the previous analyses, we used the speeding index as the dependent variable. The results supported the inverse illuminance‒speeding relationship (*b* = −0.23, *SE* = 0.09, *t* = −2.55). A likelihood ratio test comparing the full model against a model excluding illuminance showed that this comparison reached statistical significance (χ^2^ = 6.28, *p* < .05), indicating that the full model was superior and illuminance a significant predictor of speeding.

We ran two comparative analyses to analyze the extent to which illuminance may differ across the speed measurement points. First, we compared illuminance data from Zurich airport with those from an additional meteorological station located approximately 1 km east of Zurich downtown (Zurich east). For Zurich east, data were available for approximately seven months (January 1, 2007, to July 17, 2007). Correlating the illuminance measures from both stations for this timespan revealed a highly consistent pattern (*r* = .98, *p* < .001), implying that fluctuations in illuminance across geographical locations within Zurich were negligible. Second, we used illuminance data from each station separately to analyze the illuminance‒speeding relationship. Using illuminance measures from Zurich east to predict the speeding index led to similar, but slightly stronger results than did using measures from Zurich airport: As in the overall analysis, reduced illuminance was associated with increased speeding (*b* = −1.78, *t*(2404) = 8.30, *p* < .001, partial *η*^2^ = .03).

### Proxies of illuminance

We also examined proxies of illuminance and obtained converging evidence for the inverse illuminance‒speeding relationship. Specifically, the relative proportion of speeding was higher when global irradiance was lower (*b* = −4.09, *t*(27570) = −21.09, *p* < .001, partial *η*^2^ = .02), when sunshine duration was shorter (*b* = −0.07, *t*(27564) = −11.46, *p* < .001, partial *η*^2^ = .005), when sight distance was shorter (*b* = −0.09, *t*(9062) = −5.82, *p* < .001, partial *η*^2^ = .004), and when particulate air pollution was higher (*b* = 6.32, *t*(27278) = 11.83, *p* < .001, partial *η*^2^ = .005). We also found speeding to be increased under foggy (vs. clear) conditions (*b* = 7.07, *t*(6109) = 3.10, *p* = .002, partial *η*^2^ = .002), and the occurrence of fog, while being relatively rare, to be evenly distributed over time of day. Finally, as an additional control, we examined how precipitation—a condition in which drivers have been shown to slow down [2]—impacts speeding. Speeding was indeed less pronounced when precipitation was stronger (*b* = −0.15, *t*(27562) = −3.66, *p* < .001, partial *η*^2^ = .001). In conclusion, we found that—across various proxies of illuminance and contrary to the ABCs of road safety—drivers showed more speeding behavior in conditions of reduced illuminance.

## Discussion

The relationship between speeding and traffic accidents is well established [34]: A speed reduction of only 5 km/h is generally estimated to yield a 15% decrease in accidents [4]. Although the driver undoubtedly plays an important role when it comes to choosing the speed of driving [35], researchers have increasingly focused on environmental factors such as fog, contrast, and luminance. However, the results of driving simulation studies investigating the relationship between adverse visual conditions and speed have been inconclusive [19–25].

We analyzed this relationship in the field, taking advantage of a large, real-world dataset to examine whether there is a positive or negative relationship between illuminance and drivers’ speeding behavior. Across different analyses, we consistently observed an inverse relationship between illuminance and speeding, supporting the idea that a decrease in illuminance comes with an increase in speeding in an urban environment. In keeping with previous research [28], we found speeding rates to be higher at night than during the day (18.26% vs. 13.97%). Beyond differences in illuminance this day-night difference is likely to be driven by variables such as a selected subgroup of drivers, alcohol consumption, and fatigue [34–36]. Yet, it is important to note that the inverse illuminance–speeding relationship also emerged when we focused solely on daytime data (e.g., measurements between 12 noon and 1 pm; Fig 2). These results can be explained by the Thomson effect [17], which postulates that reduced contrast (operationalized through illuminance in our setup) leads to an underestimation of speed. In situations of reduced contrast, drivers may increase their speed unintentionally due to a biased perceptual input. However, we cannot rule out that other factors are at play as well, such as different affective responses or wakefulness due to varying illuminance levels. An important note is also that other research [23–25] has found contradicting results in lab settings. The truth may lie between the highly controlled, yet artificial lab setting and the less controlled, yet more natural field setting.

The current examination of the illuminance‒speeding relationship is the first to make use of a vast sample (i.e., over 1.2 million observations). The resulting findings add to the growing list of environmental factors that can bias human perception [37] and demonstrate that, in contrast to prior research that mainly focused on average speeds, illuminance also affects the likelihood and risk of exceeding the legal speed limit. This view is supported by field research on accident prevention showing that both more natural light (as examined in the context of daylight saving time [38,39]) and more artificial light (as examined by manipulating the illuminance of road lighting [40,41]) can result in fewer traffic accidents and fatalities. Our analyses suggest a mechanism underlying this illuminance‒accident relationship: drivers’ increased tendency to speed when illuminance is reduced. More generally, our approach shows that large datasets can be used not only for data mining and to explore unknown regularities [42] but also to provide real-world evidence of a hypothesized relationship that could not be tested conclusively in laboratory settings.

Notwithstanding the many benefits of a large, real-world dataset, limitations exist. The roads monitored in our dataset met planning and operational demands and hence do not represent a random sample of all Zurich roads. They are also urban and in parts speed-reduced; the degree to which an inverse illuminance‒speeding relationship can be found at higher speeds (e.g., on highways) remains an open question, especially in view of research suggesting that the Thompson effect may be attenuated in such conditions [16]. Another limitation is that the location of illuminance measurement was not identical with the location of speed measurement, although differences in illuminance across measurement points were shown to be negligible. Finally, we did not experimentally manipulate illuminance and thus cannot isolate the cause of its relationship with speeding. Future research may therefore address several issues. A follow-up field study could measure illuminance and speed at the same location (to ensure that drivers are exposed to the same conditions as the measurement instruments) and examine a larger speed distribution (beyond the maximum speed of 50 km/h in our sample). Moreover, it would be promising to test the above issues in a controlled field experiment that allows to vary illuminance in a systematic manner. One could achieve this in a tunnel where no external light sources or ambient light interfere with the measurements, providing causal evidence for the issues at hand. The underlying mechanism could be tested by individual-level data based on objective (e.g., vehicle headlights, blood alcohol level, and fatigue) and subjective measurements (e.g., inquiring drivers about their motives to speed).

Although the reported effects are subtle in statistical terms, they are potentially vital from a policy perspective because they play out across countless vehicle movements. Specifically, they suggest two distinct interventions that may reduce the frequency of speeding. One is information: Drivers should be educated (e.g., in driving schools) about the counterintuitive relationship between reduced illuminance and the inclination to speed—just as they are instructed about the effects of fatigue on reaction time. Also, drivers could be informed (e.g., via built-in warning systems or smartphone apps) about particularly hazardous situations such as poor visual conditions under fog. Information and education, however, may not suffice if the effect of illuminance works on a perceptual level that is difficult to access through heightened awareness. Therefore, part of the remedy may be delegated to the environment rather than the individual. Specifically, changes in environmental illuminance might be compensated for by equipping vehicles with higher intensity headlights. Alternatively, policy makers could opt to install smart illuminance-dependent road lighting that is aligned with the functionality of headlights. Such solutions, however, may exact substantial expenses and the risk of unintended side effects. For instance, according to the notion of risk compensation [43], drivers may compensate for smart road lighting through increased speed and reduced concentration, which could undo any accident-reducing effect. In the long term, other technological solutions—such as autonomous cars—are conceivable that will not only address the inverse illuminance‒speeding relationship but also attenuate other traffic risks caused by the regularities of the human perceptual system and its inescapable bounds.

## Supporting information

S1 Fig Location of monitored roads across Zurich. The Traffic Division of the City of Zurich regularly uses hidden radar systems to measure the speed of passing vehicles for city planning purposes. Our analyses are based on the measured speed of 1,220,359 vehicle movements collected in 71 urban roads (see red dots).
(PNG)

S2 Fig Speeding over a 24-hour period. We calculated a speeding index by dividing the number of vehicles exceeding the speed limit by the total number of vehicles (per road and hour). Time of day corresponds to the local time (UTC+1 adjusted for daylight saving time). The figure shows the mean daily fluctuations in speeding, with the highest speeding rates occurring in the early morning hours.
(PNG)

S3 Fig Illuminance over a 24-hour period and across seasons. We calculated the average illuminance (log[lux]) in Zurich for each hour of the day (between 2007 and 2009) split into four seasons. Time of day corresponds to the local time (UTC+1 adjusted for daylight saving time). The figure shows the mean daily fluctuations in illuminance, from minimal illuminance values in the early morning hours to maximum illuminance shortly after noon. Seasonal changes in illuminance are apparent in the earlier increase in illuminance in the morning (summer < spring < fall < winter) and the later decrease in illuminance in the evening (summer > spring > fall > winter).
(PNG)

S1 Table Sample size and characteristics of 71 roads. (DOCX)
