# Supplementary figures and images for: Blind haste: As light decreases, speeding increases
Source: PLoS One. 2018 Jan 3;13(1):e0188951. doi: 10.1371/journal.pone.0188951 (PMC5751981; doi:10.1371/journal.pone.0188951)

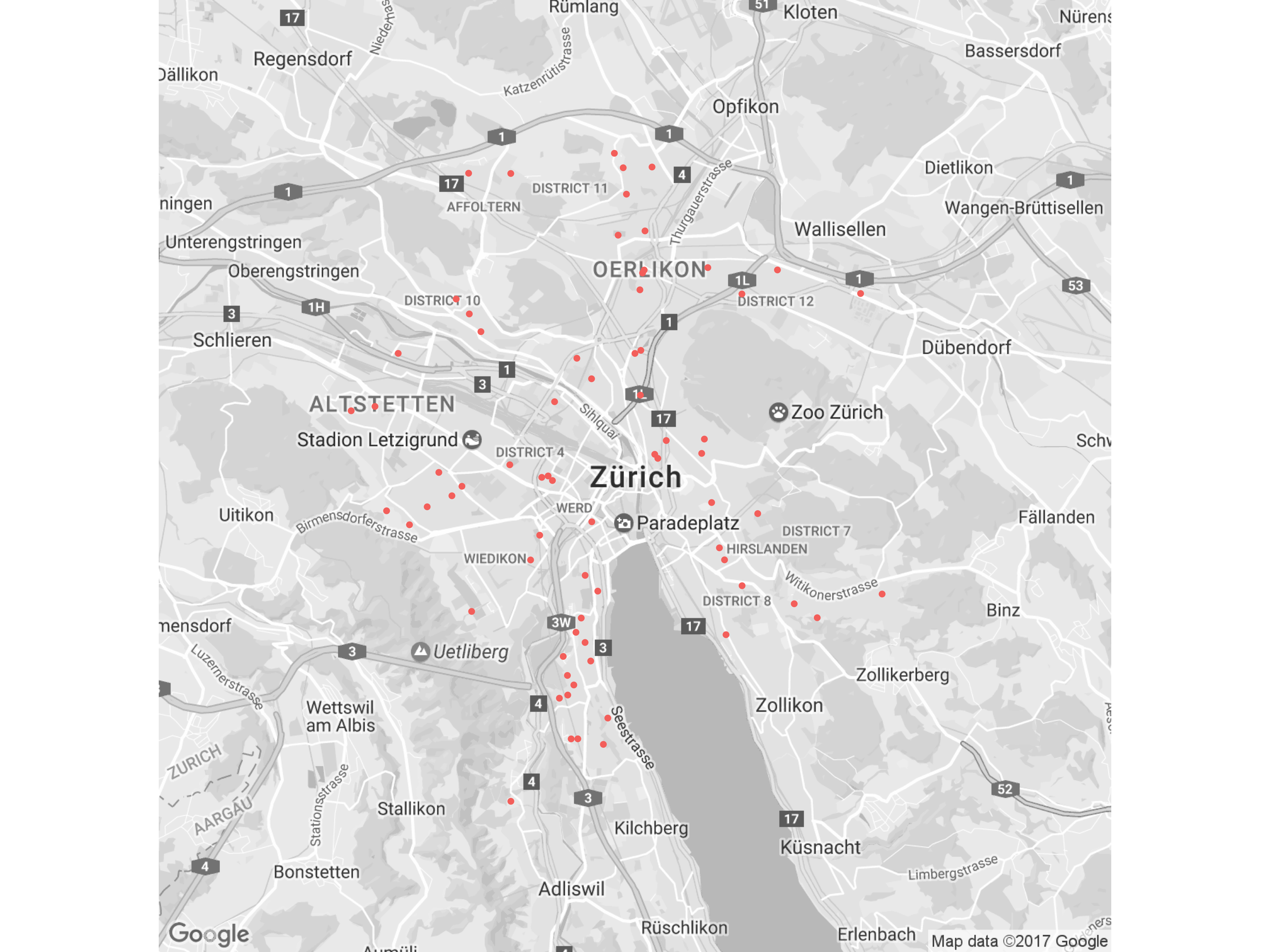

Supplement: S1 Fig — The Traffic Division of the City of Zurich regularly uses hidden radar systems to measure the speed of passing vehicles for city planning purposes. Our analyses are based on the measured speed of 1,220,359 vehicle movements collected in 71 urban roads (see red dots). (PNG) [file pone.0188951.s001.png]

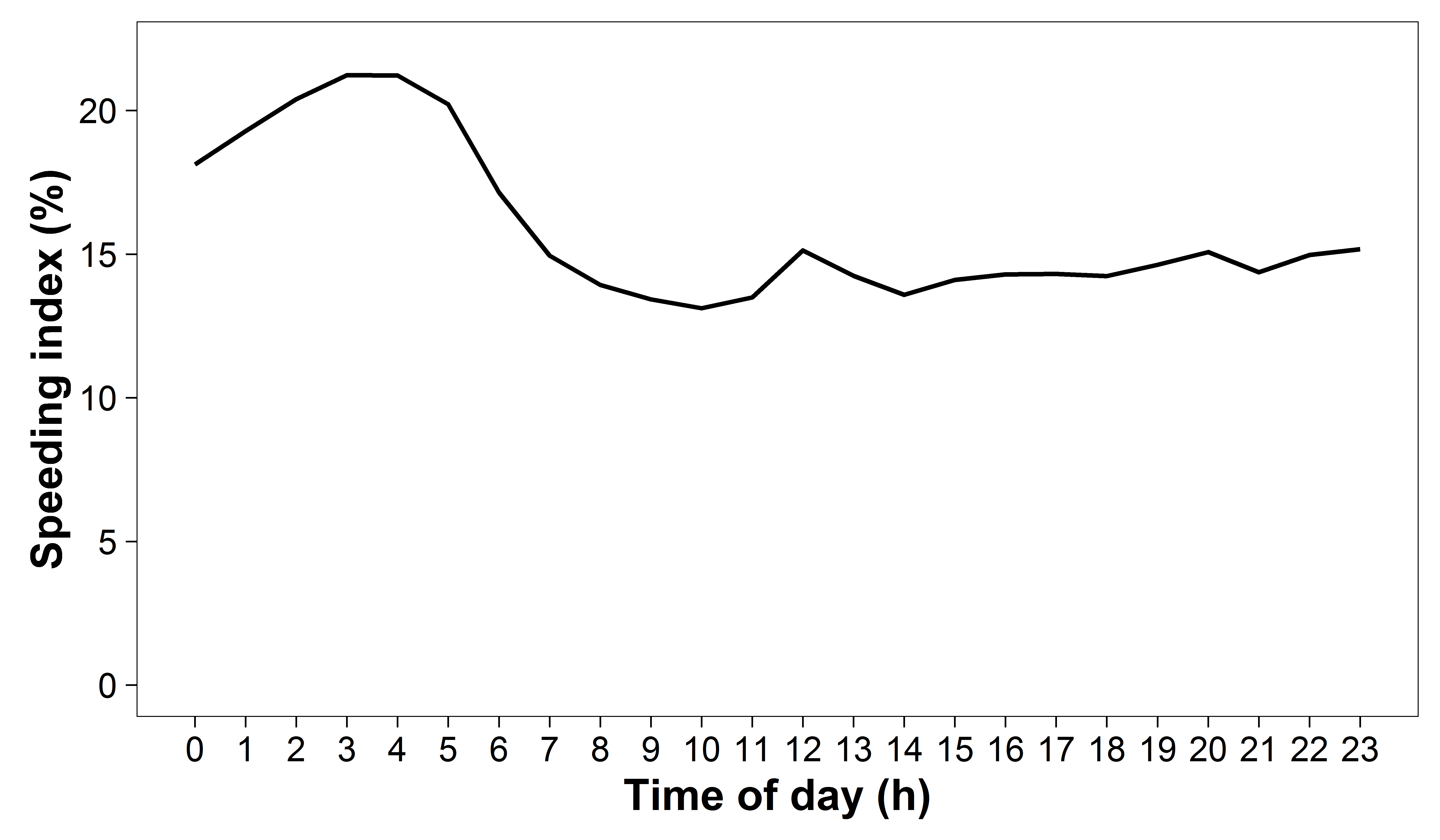

Supplement: S2 Fig — We calculated a speeding index by dividing the number of vehicles exceeding the speed limit by the total number of vehicles (per road and hour). Time of day corresponds to the local time (UTC+1 adjusted for daylight saving time). The figure shows the mean daily fluctuations in speeding, with the highest speeding rates occurring in the early morning hours. (PNG) [file pone.0188951.s002.png]

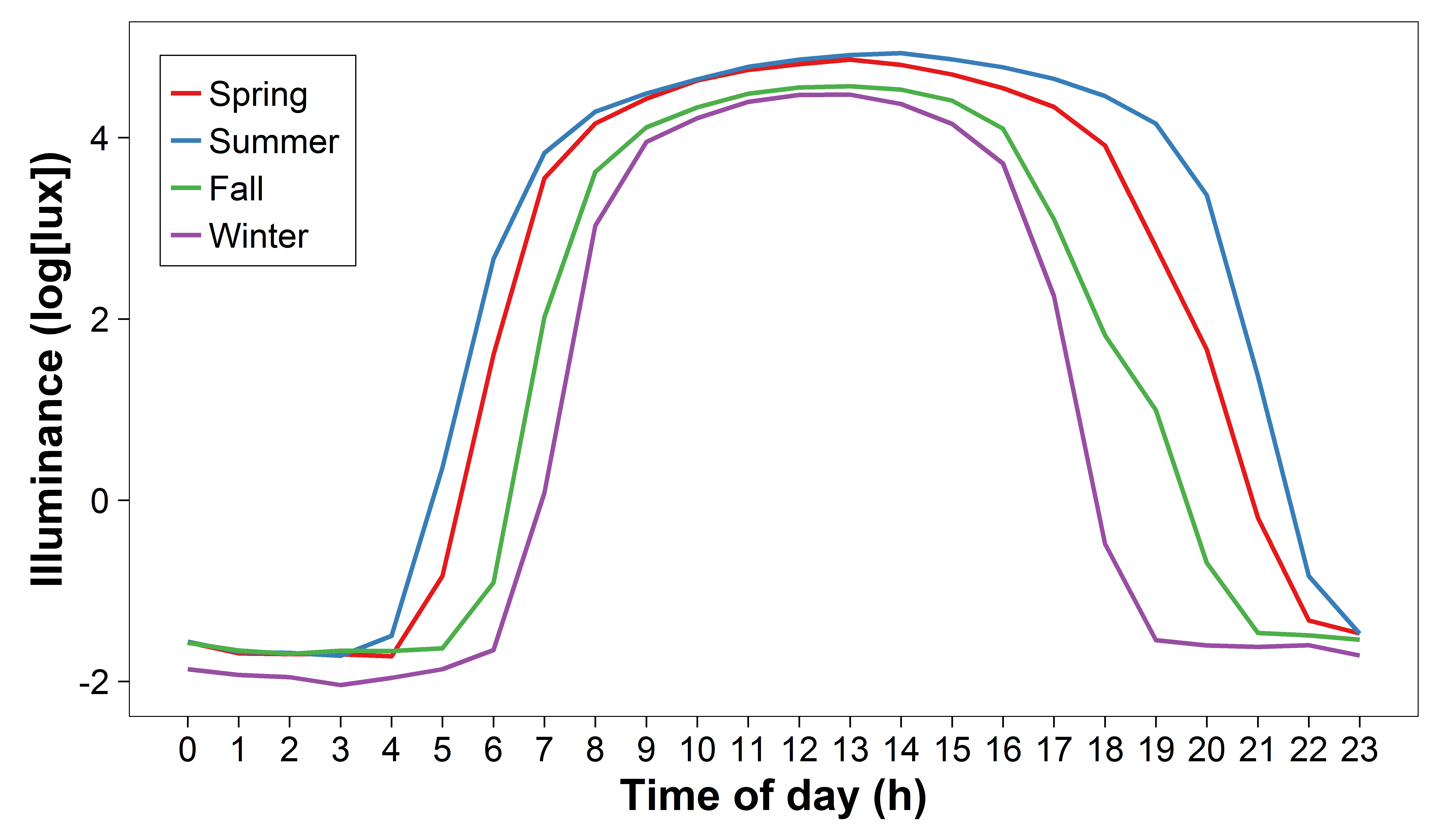

Supplement: S3 Fig — We calculated the average illuminance (log[lux]) in Zurich for each hour of the day (between 2007 and 2009) split into four seasons. Time of day corresponds to the local time (UTC+1 adjusted for daylight saving time). The figure shows the mean daily fluctuations in illuminance, from minimal illuminance values in the early morning hours to maximum illuminance shortly after noon. Seasonal changes in illuminance are apparent in the earlier increase in illuminance in the morning (summer < spring < fall < winter) and the later decrease in illuminance in the evening (summer > spring > fall > winter). (PNG) [file pone.0188951.s003.png]
