# Supplementary material for: Blind haste: As light decreases, speeding increases
Source: PLoS One. 2018 Jan 3;13(1):e0188951. doi: 10.1371/journal.pone.0188951 (PMC5751981; doi:10.1371/journal.pone.0188951)
Supplement: S1 Table — (DOCX) [file pone.0188951.s004.docx]

**S1 Table. Sample size and characteristics of 71 roads.**

| Road Name | Measurement Frequency^a^ | No. of Vehicles | Type of Area | Speed Limit (km/h) | Road Alignment^b^ | No. of Lanes^c^ | Distance to Closest Crossroad (m)^d^ |
| --- | --- | --- | --- | --- | --- | --- | --- |
| Agnesstrasse | 1 | 2819 | urban | 30 | straight | 1 | 90 |
| Am Oeschbrig | 1 | 2151 | urban | 30 | curved | 1 | 30 |
| Armin-Bollinger-Weg | 1 | 8105 | urban | 30 | straight | 1 | 50 |
| Attenhoferstrasse | 1 | 4016 | urban | 30 | straight | 1 | 30 |
| Ausserdorfstrasse | 1 | 5522 | urban | 30 | straight | 1 | 20 |
| Bachtobelstrasse | 1 | 24391 | rural | 30 | straight | 1 | 180 |
| Baendlistrasse | 1 | 11857 | urban | 30 | straight | 1 | 120 |
| Baumackerstrasse | 1 | 7621 | urban | 30 | straight | 1 | 30 |
| Bellariastrasse | 2 | 7885 | urban | 30 | straight | 1 | 100 |
| Conrad-Ferdinand-Meyer-Strasse | 1 | 10131 | urban | 30 | straight | 1 | 30 |
| Culmannstrasse | 2 | 22760 | urban | 30 | straight | 1 | 40 |
| Drusbergstrasse | 1 | 2204 | urban | 30 | curved | 2 | 20 |
| Eidmattstrasse | 1 | 15266 | urban | 30 | straight | 1 | 30 |
| Feldblumenstrasse | 1 | 8393 | urban | 30 | straight | 1 | 30 |
| Feldstrasse | 1 | 4319 | urban | 30 | straight | 1 | 50 |
| Fellenbergstrasse | 2 | 70840 | urban | 50 | straight | 2 | 100 |
| Ferdinand-Hodler-Strasse | 1 | 2257 | urban | 30 | straight | 1 | 190 |
| Floessergasse | 1 | 18310 | urban | 30 | straight | 1 | 50 |
| Freiestrasse | 1 | 12038 | urban | 30 | straight | 1 | 90 |
| Freudenbergstrasse | 1 | 8398 | urban | 30 | straight | 2 | 70 |
| Frohalpstrasse | 1 | 2870 | urban | 30 | straight | 1 | 130 |
| Geibelstrasse | 1 | 1870 | urban | 30 | straight | 1 | 100 |
| Girhaldenstrasse | 1 | 5565 | urban | 30 | straight | 1 | 40 |
| Gubelstrasse | 1 | 11294 | urban | 30 | straight | 1 | 80 |
| Gutstrasse | 1 | 46504 | urban | 30 | straight | 2 | 120 |
| Hagenbuchrain | 1 | 194 | transition | 30 | curved | 1 | > 200 |
| Haldenstrasse | 2 | 64464 | urban | 30 | straight | 2 | 70 |
| Hammerstrasse | 1 | 3042 | urban | 30 | straight | 1 | > 200 |
| Haumesserstrasse | 1 | 2214 | urban | 30 | straight | 1 | 80 |
| Heinrichstrasse | 1 | 188402 | urban | 30 | straight | 2 | 170 |
| Hoehenring | 1 | 3605 | urban | 30 | curved | 1 | 80 |
| Holzwiesweg | 1 | 1811 | urban | 30 | straight | 1 | 170 |
| Huttenstrasse | 1 | 4446 | urban | 30 | straight | 1 | 20 |
| Im Maas | 1 | 845 | urban | 30 | straight | 1 | 60 |
| Jungholzstrasse | 1 | 11155 | urban | 30 | straight | 1 | 130 |
| Kalchbuehlstrasse | 2 | 98655 | transition | 30/50 | straight | 2 | 160 |
| Kanzleistrasse | 1 | 15790 | urban | 30 | straight | 1 | 170 |
| Kilchbergstrasse | 1 | 5349 | transition | 30 | curved | 2 | 100 |
| Klebestrasse | 1 | 10377 | urban | 30 | straight | 1 | 90 |
| Koeschenruetistrasse | 1 | 21633 | urban | 30 | straight | 1 | > 200 |
| Kuerbergstrasse | 1 | 21218 | urban | 30 | straight | 1 | 70 |
| Kurfirstenstrasse | 1 | 865 | transition | 30 | straight | 1 | 120 |
| Letzigraben | 3 | 132673 | urban | 30/50 | straight | 1/2 | 120 |
| Luegislandstrasse | 3 | 40390 | transition | 30 | straight | 2 | 100 |
| Marchwartstrasse | 1 | 1290 | transition | 30 | straight | 1 | 80 |
| Moosstrasse | 1 | 2208 | urban | 30 | straight | 1 | 100 |
| Morgentalstrasse | 1 | 9506 | urban | 30 | curved | 1 | 100 |
| Muehlackerstrasse | 1 | 15360 | rural | 30 | straight | 2 | 50 |
| Nordstrasse | 1 | 41320 | urban | 50 | straight | 1 | 160 |
| Pflanzschulstrasse | 1 | 4794 | urban | 30 | straight | 2 | 70 |
| Rainstrasse | 2 | 1470 | transition | 30 | straight | 1 | 100 |
| Riedgrabenweg | 2 | 23528 | transition | 30 | straight | 2 | 80 |
| Roeslistrasse | 1 | 3547 | urban | 30 | curved | 1 | 70 |
| Rossackerstrasse | 1 | 1425 | transition | 30 | straight | 1 | 200 |
| Scheideggstrasse | 1 | 5497 | urban | 30 | straight | 1 | 80 |
| Scheuchzerstrasse | 2 | 25926 | transition | 30 | straight | 1 | 50 |
| Schulhausstrasse | 2 | 28124 | transition | 30 | straight | 1 | 80 |
| Schwellistrasse | 1 | 1739 | transition | 30 | curved | 2 | 100 |
| Sonneggstrasse | 1 | 28412 | urban | 30 | straight | 2 | 120 |
| Sonnenbergstrasse | 1 | 9430 | rural | 30 | curved | 2 | 120 |
| Speerstrasse | 1 | 1311 | urban | 30 | straight | 2 | 180 |
| Staubstrasse | 1 | 2393 | urban | 30 | straight | 1 | 50 |
| Stuessistrasse | 1 | 4909 | urban | 30 | straight | 1 | 50 |
| Thujastrasse | 1 | 2491 | urban | 30 | straight | 1 | 50 |
| Toblerstrasse | 1 | 1599 | urban | 30 | straight | 1 | 50 |
| Tulpenstrasse | 1 | 1771 | urban | 30 | straight | 2 | 90 |
| Ueberlandstrasse | 1 | 3193 | transition | 50 | straight | 2 | 30 |
| Venusstrasse | 1 | 635 | urban | 30 | straight | 1 | 70 |
| Wehrenbachhalde | 1 | 4463 | transition | 30 | straight | 1 | 120 |
| Wolfswinkel | 1 | 39079 | transition | 30 | straight | 2 | 100 |
| Zollikerstrasse | 1 | 10425 | transition | 30 | straight | 2 | 50 |

^a^ A total of eleven roads served as measurement point more than once.

^b^ We coded road alignment as “straight” if there was no significant curve for at least 50 m in both directions from the measurement point.

^c^ Most roads are two-lane roads (one lane per direction). However, especially in 30 km/h zones, the lanes are not always visually distinguishable.

^d^ In the case of several measurement points per road, we reported the average distance to the closest crossroad. The distance to the closest crossroad with traffic lights was larger than 200 m for all roads, with one exception (Ueberlandstrasse: 100 m).
